# Application of Machine Learning Classifiers in Rapid Reviews for Health Research: A Case Example Using EPPI‐Reviewer

**DOI:** 10.1002/cesm.70101

**Published:** 2026-08-10

**Authors:** Sarah R. Prowse, Zak Ghouze, Shaun Treweek

**Affiliations:** ^1^ Aberdeen Centre for Evaluation University of Aberdeen Aberdeen UK; ^2^ EPPI‐Centre Social Science Research Unit, UCL Institute of Education University College London London UK; ^3^ Department of Women's and Children's Health Uppsala University Uppsala Sweden

**Keywords:** clinical trial, data mining, information storage and retrieval, machine learning, review

## Abstract

**Introduction:**

Rapid reviews aim to deliver timely evidence for decision‐makers when full systematic reviews are not possible or practical. Efficient selection of studies is challenging when questions are complex, or the evidence base is diffuse.

**Methods:**

In a rapid review on trial informativeness, our team used EPPI‐Reviewer, a web‐based systematic review platform that supports document management, screening, and machine learning prioritization, to conduct title and abstract screening. We developed a machine learning classifier model within the platform to rank records by predicted relevance based on coding structures aligned with predefined criteria.

**Results:**

The classifier model correctly concentrated relevant studies in the higher probability bands, which allowed most eligible records to be identified early. As screening progressed to lower probability bands, the number of newly identified records declined, indicating effective prioritization. Real‐time collaboration and a clear audit trail supported consistent decision‐making across reviewers. Limitations included the initial effort to train the model and potential subscription costs.

**Conclusion:**

Classifier assisted screening in EPPI‐Reviewer improved the feasibility of conducting a rapid review on a complex topic within a limited timeframe. Although the risk of missed citations remains, this is inherent to any review method. With appropriate training and support, classifier models and platforms like EPPI‐Reviewer can enhance both efficiency and transparency in rapid evidence synthesis.

## 1 Introduction

Rapid reviews are increasingly commissioned by policymakers and health organizations that require timely evidence to inform urgent social and care decisions [1]. Unlike traditional systematic reviews, rapid reviews adapt or streamline certain processes to balance rigor with speed [1, 2]. These adaptations allow for rapid synthesis when research topics are complex, evidence is diffuse, or resources for a full review are limited or not available [2]. However, the need for efficiency can place greater demands on review teams, particularly during study selection, where large numbers of citations or records must be screened within a limited timeframe.

To address this challenge, many systematic review platforms now use machine learning tools that prioritize likely relevant records during the screening process [3, 4]. A classifier model is a type of machine learning algorithm that learns from the inclusion and exclusion decisions made by reviewers to predict the probability that unscreened records will be relevant [5]. By reducing the number of records that must be screened or read in full, these models can potentially accelerate the review process without compromising transparency.

Our wider team conducted a rapid review of global insights on trial informativeness, published in June 2025 [6]. Trial informativeness refers to the extent in which a clinical trial is designed, conducted, and reported in ways that ensure its results are useful for decision‐making and future research [7]. In our rapid review, we used EPPI‐Reviewer, a web‐based systematic review platform, to manage title and abstract screening using a classifier model [8]. This communication reflects on the experience and outlines how EPPI‐Reviewer can support timely evidence generation in a rapid review context. We highlight the strengths and limitations encountered and consider the implications for teams undertaking rapid reviews of similarly complex questions.

## 2 Methods

The term “informativeness,” coined by Zarin and colleagues, is not yet widespread in the lexicon of trials research which made developing a search strategy and retrieving initial records challenging [7]. Our rapid review sought to synthesize literature on how to ensure clinical trials produce evidence that is both useful and relevant, an area characterized by broad scope and heterogenous terminology. Several search strategies were designed and trialed with the assistance of an information specialist, eventually resulting in 9145 records for further screening. Searching and screening details for the rapid review are available on PROSPERO (record no.: CRD42024541229) [9].

To manage the large volume of retrieved citations within a limited 3‐month timeframe, we used EPPI‐Reviewer (version 6), a web‐based systematic review platform developed by the Evidence for Policy and Practice Information (EPPI) Centre at University College London. Collaboration was sought with a member of the EPPI‐Reviewer team [ZG], who further advised on the appropriate use of coding structures, application of the classifier model, and the general use of EPPI‐Reviewer's web‐based interface.

After importing retrieved citations to the platform, we developed and applied a classifier model in EPPI‐Reviewer to support title and abstract screening. The classifier model operates by learning from reviewer decisions and then ranking unscreened records by probability of relevance. We began with a training set of *n* = 1822 records (20%, after the removal of duplicate records, *n* = 36) screened by a single reviewer [SP] to establish an initial model. Screening decisions were recorded using coding structures aligned with our predefined inclusion and exclusion criteria. High‐probability records identified when the classifier model was applied were prioritized for review, allowing us to source potentially eligible studies earlier in the screening process.

## 3 Results and Reflections

A total of 8812 records (97%) were excluded after the application of the classifier model. Records with a greater than 50% probability of inclusion were subject to further screening (*n* = 297). An additional 10% manual screening was undertaken by two reviewers [SP and ST] of citations placed by EPPI‐Reviewer in the lower probability bands of 40%–50% and 30%–40%, where agreement was reached that no relevant records had been excluded, and that EPPI‐Reviewer was identifying citations appropriately. Screening decisions were logged within EPPI‐Reviewer, creating a transparent audit trail. The final rapid review included 42 texts for analysis either identified through manual screening, or as high probability records for inclusion by the EPPI‐Reviewer platform [6].

**FIGURE 1:**
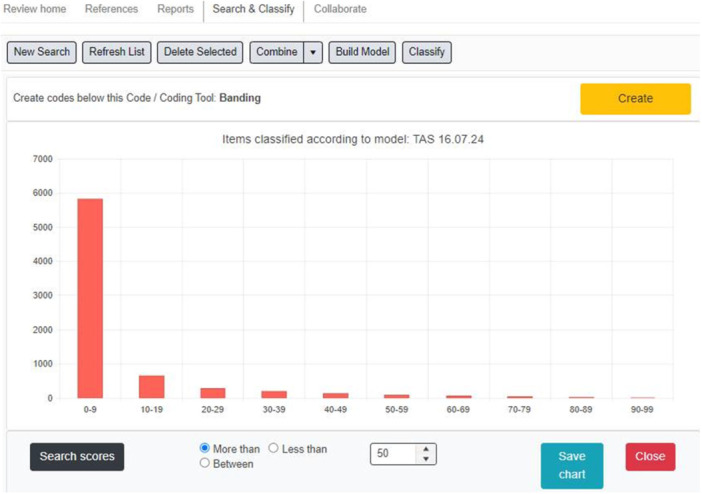
The screening yield curve peaks in the low‐probability bands, reflecting that most records were accurately predicted by the EPPI‐Reviewer platform to be irrelevant.

Using EPPI‐Reviewer for title and abstract screening allowed us to manage a large number of records within the limited 3‐month timeframe of our rapid review. The classifier model was essential when balancing speed with rigor. After training the system on an initial 20% of manually screened records, the model reliably ranked those studies likely to be relevant in probability bands of 50% or greater. The screening yield curve seen in Figure 1 illustrates this effect where most records are noted in the lowest probability band of 0%–9% when the classifier model was applied, meaning the vast majority of citations would not be aligned with the focus of our rapid review.

New users may face a learning curve in navigating the EPPI‐Reviewer interface, especially when setting up coding structures and understanding how a classifier model is designed and applied. Appropriate training is critical to realizing the benefits of the EPPI‐Reviewer platform, of which the EPPI‐Centre has developed numerous supporting resources [10, 11]. In our experience, compared with other screening tools such as Rayyan or Covidence, EPPI‐Reviewer provided stronger systems for evidencing transparency, reproducibility, and auditability. However, lighter platforms may remain appropriate for more narrowly defined questions or for teams seeking minimal barriers to entry. Subscription costs may also be prohibitive for smaller groups or those without institutional licenses, although EPPI‐Reviewer is currently free to trial on a limited time basis with discounted rates for those working in low‐to‐middle income countries. EPPI‐Reviewer is a not‐for‐profit service, and fees contribute to costs associated with infrastructure, development, and support of the software. Direct support is included with user accounts, and the EPPI‐Centre have a small team of support staff available to answer user queries.

Our experience using EPPI‐Reviewer's classifier modeling capabilities for title and abstract screening in a rapid review highlighted both the potential and the current limitations of machine learning approaches in evidence synthesis. The main benefit we observed was efficiency. By prioritizing records most likely to be relevant, the classifier model enabled us to work through a large dataset within a limited timeframe but with high confidence that relevant records were not being missed. This was valuable for a complex and conceptually diffuse question such as trial informativeness, where traditional screening could have delayed progress. There is always a risk of missed citations when using a classifier model, although this limitation is consistent with the broader compromises inherent in rapid review methods. Current research has shown the use of classifiers results in very high recall (i.e., most eligible records are retrieved) with few citations incorrectly assigned (i.e., high precision) [12, 13].

As this work was undertaken in the context of a rapid review, methodological decisions were made to balance rigor with the practical constraints of accelerated timelines and available resources. A separate validation and calibration set was not used, which limits the extent to which classifier performance can be assessed beyond this specific review context. In addition, use of a single screener for the training set may have introduced reviewer‐specific decision‐making patterns into the model.

Machine learning is not a substitute for methodological expertise, but when embedded within a structured platform it can make rapid evidence production more feasible without sacrificing scientific rigor. Overall, our experience was that the classifier model meaningfully reduced the workload associated with screening while maintaining methodological rigor. Future adoption of platforms like EPPI‐Reviewer for rapid reviews will depend heavily on training, support, and financial access, but the potential exists to further improve the feasibility of rapid reviews for complex or diffuse health topics.

## Author Contributions

**Sarah R. Prowse:** conceptualization, investigation, writing – original draft, methodology, formal analysis, validation, project administration, resources. **Zak Ghouze:** writing – review and editing, software. **Shaun Treweek:** conceptualization, funding acquisition, writing – review and editing, methodology, validation, supervision.

## Ethics Statement

Ethics approval was not required, as no human participants or new data collection were involved.

## Conflicts of Interest

One author [Z.G.] is employed by the EPPI‐Centre and contributed expertise regarding software functionality. The other authors declare no conflicts of interest.

## Data Availability

This article reports methodological reflections and does not generate new datasets. All screening data related to the rapid review by Prowse et al. (2025) are contained within the original publication. No additional data are available.
